# Prediction-learning in infants as a mechanism for gaze control during object exploration

**DOI:** 10.3389/fpsyg.2014.00441

**Published:** 2014-05-20

**Authors:** Matthew Schlesinger, Scott P. Johnson, Dima Amso

**Affiliations:** ^1^Department of Psychology, Southern Illinois University Carbondale, CarbondaleIL, USA; ^2^Department of Psychology, University of California Los Angeles, Los AngelesCA, USA; ^3^Cognitive, Linguistic, and Psychological Sciences, Brown University, ProvidenceRI, USA

**Keywords:** object perception, prediction-learning, infant development, eye movements, visual saliency

## Abstract

We are pursuing the hypothesis that visual exploration and learning in young infants is achieved by producing gaze-sample sequences that are sequentially predictable. Our recent analysis of infants’ gaze patterns during image free-viewing (Schlesinger and Amso, 2013) provides support for this idea. In particular, this work demonstrates that infants’ gaze samples are more easily learnable than those produced by adults, as well as those produced by three artificial-observer models. In the current study, we extend these findings to a well-studied object-perception task, by investigating 3-month-olds’ gaze patterns as they view a moving, partially occluded object. We first use infants’ gaze data from this task to produce a set of corresponding center-of-gaze (COG) sequences. Next, we generate two simulated sets of COG samples, from image-saliency and random-gaze models, respectively. Finally, we generate learnability estimates for the three sets of COG samples by presenting each as a training set to an SRN. There are two key findings. First, as predicted, infants’ COG samples from the occluded-object task are learned by a pool of simple recurrent networks faster than the samples produced by the yoked, artificial-observer models. Second, we also find that resetting activity in the recurrent layer increases the network’s prediction errors, which further implicates the presence of temporal structure in infants’ COG sequences. We conclude by relating our findings to the role of image-saliency and prediction-learning during the development of object perception.

## INTRODUCTION

The capacity to perceive and recognize objects begins to develop shortly after birth (e.g., Fantz, 1956; Slater, 2002). A critical skill that emerges during this time and supports object perception is gaze control, that is, the ability to direct gaze toward informative or distinctive regions of an object, such as edges and contours, as well as to shift gaze from one part of the object to another (e.g., Haith, 1980; Bronson, 1982, 1991). There are a number of relatively well-studied mechanisms that help drive the development of gaze control – in particular, during infants’ visual object exploration – including improvements in acuity and contrast perception, inhibition-of-return, and selective attention (e.g., Banks and Salapatek, 1978; Clohessy et al., 1991; Dannemiller, 2000). While these mechanisms help to explain when, why, and in which direction infants shift their gaze, they may offer limited explanatory power in accounting for gaze-shift patterns at a more fine-grained level (e.g., the particular visual features sampled by the fovea at the next fixation point).

In the current paper, we present and evaluate a microanalytic approach for analyzing infants’ gaze shift sequences during visual exploration. Specifically, we convert the sequence of fixations produced by each infant into a stream of “center-of-gaze” (or COG) image samples, where each sample approximates the portion of the image visible to the fovea of a human observer while fixating the given location on the image (for a related approach, see Dragoi and Sur, 2006; Kienzle et al., 2009; Mohammed et al., 2012). We then use a simple recurrent network (SRN) as a computational tool for estimating the presence of temporal or sequential structure within infants’ COG gaze patterns.

The rationale for our analytical strategy is guided by two key ideas: first, that a core learning mechanism in infancy is driven by the detection of statistical regularities in the environment (e.g., Saffran et al., 1996), and second, that a wide range of infants’ exploratory actions, such as visual scanning and object manipulation, are future-oriented (e.g., Haith, 1994; Johnson et al., 2003; von Hofsten, 2010). Together, these ideas suggest that infants’ ongoing gaze patterns are predictive or prospective. Thus, our primary hypothesis is that if infants’ gaze patterns are sequentially structured, we should then find that the stream of recent fixations toward an object or scene will provide sufficient information to predict the content of upcoming fixations. A related hypothesis is, given that sequential structure is observed in infants’ gaze patterns, these sequences should be more predictable (i.e., more easily learned by an SRN) than those generated by other types of observers (e.g., human adults, ideal, or artificial observers, etc.).

Our recent work has provided preliminary support for both of these hypotheses. In particular, we compared the gaze sequences produced by 3-month-old infants and adults during an image free-viewing task with those from three sets of artificial observers (i.e., image-saliency, image-entropy, and random-gaze models) that were presented with the same natural images (Schlesinger and Amso, 2013; Amso et al., 2014). The real and artificial observers’ fixation data were first transformed into corresponding sequences of COG samples. We then measured the learnability of the five sets of COG image sequences by presenting each set to an SRN, which was trained to reproduce the corresponding sequences. A key finding from this work, over two simulation studies, was that the COG sequences produced by the human infants resulted in both more accurate and rapid learning than the adult COG sequences, or any of the three artificial-observer sequences.

**FIGURE 1:**
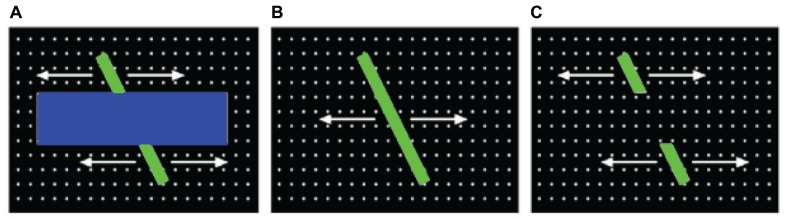
**Displays used to assess perceptual completion in infants: (A) occluded-rod (habituation) display, and (B) complete-rod and (C) broken-rod test displays**.

In the current paper, we extended our model in a number of important ways to investigate the development of object perception in 3-month-olds. First, our dataset derives from a paradigm called the *perceptual-completion task*, which is specifically designed to assess infants’ perception of a moving, partially occluded object (Kellman and Spelke, 1983; Johnson and Aslin, 1995). **Figure 1A** illustrates this occluded-rod display, which is presented first to infants, and then repeated until they habituate to the display. Two subsequent displays are then presented to infants and used to probe their perception and memory of the occluded-rod display (see **Figures 1B,C**). Because our focus here is on infants’ initial gaze patterns at the beginning of the task, before they have accumulated extensive experience with the display, we therefore restrict our analyses to gaze data from the first trial of the occluded-rod display. Although this display is somewhat simplified relative to the natural images from our previous study, it also has the benefit that infants will likely devote much of their attention to either of the two primary objects in the scene (i.e., the moving rod and/or the occluder), thereby producing a rich source of object-directed gaze data to analyze.

A second important advance in the current paper concerns how the artificial-observer gaze patterns are produced. Specifically, in our previous model, several parameters of the artificial observers were left to vary freely, which resulted in systematic differences between the kinematics of the gaze patterns produced by the human-infant and artificial observers. For example, the artificial observers generated significantly longer gaze shifts than the infants. We address this issue in the current model by carefully yoking the gaze patterns of each artificial observer to a corresponding individual infant, so that the average kinematic measures were the same for each observer group.

A third advance is that we also simplified the architecture of the model used to learn the COG sequences. In particular, our previous model focused specifically on the process of visual exploration, including a component in the model that simulated an *intrinsically motivated learner* (i.e., an agent that is motivated to improve its own behavior, rather than to reach an externally defined goal). However, because the issue of intrinsic motivation is not central to the current paper, we have stripped this component from the model, resulting in a more direct and straightforward method for assessing the relative learnability of the COG sequences produced by each of the observer groups.

In the next section, we provide a detailed description of (1) the procedure used to transform infants’ gaze data into COG sequences, (2) the comparable steps used to generate the artificial observers’ gaze data and COG sequences, and (3) the training regime employed to measure COG sequence learnability. In the meantime, we briefly sketch the procedure here, followed by our primary hypotheses and analytical strategy.

The infant gaze data were obtained from a sample of 3-month-old infants who viewed the occluded-rod display illustrated in **Figure 1A**. Fixation locations for each infant were acquired by an automated eye-tracker. These locations were then mapped to the corresponding spatial position and frame number from the occluded-rod display, and a small (41 × 41 pixel) image sample, centered at the fixation location, was obtained for each gaze point. Next, two sets of artificial gaze sequences were generated. First, an image-saliency model was used to produce a sequence of gaze points in which gaze direction is determined by bottom-up visual features, such as motion or regions with strong light/dark contrast (e.g., Itti and Koch, 2000). Second, in the random-gaze model, locations were selected at random from the occluded-rod display. Each of the artificial-observer models was used to generate a set of COG sequences, with each sequence in the set yoked to the timing and gaze-shift distance of a corresponding infant.

Given our previous findings with the image free-viewing paradigm, our primary hypothesis was that the COG sequences produced by infants during the occluded-rod display would be more easily learned by a set of SRNs than either of the two artificial-observer sequences. We evaluated this hypothesis by assigning an SRN to each of the infants, and then training each network simultaneously on the three corresponding COG sequences (i.e., the infant’s sequence, plus the yoked image-saliency and random-gaze sequences). Learning was implemented in each SRN by presenting it with the three corresponding COG sequences, one image sample at a time as input, and then using a supervised learning algorithm to train the SRN to produce as output the next image sample from the sequence. We then assessed *learnability* by ranking the three observers assigned to each SRN by mean prediction error after each training epoch. Given this measure, we predicted that infants would not only have the highest average rank at the start of training (i.e., their COG sequences would be learned first by the SRNs), but also that this difference would persist throughout training.

In addition, we also probed the training process further by exploring the effect of manipulating the context units on the performance of the SRN. In particular, we implemented a “forgetting function” in which the context units were reset at one of three intervals (every 1, 2, or 5 COG training samples; for a related discussion, see Elman, 1993). In the most extreme condition, resetting the context units after each COG sample enabled us to determine if the network was learning exclusively on the basis of each current COG sample – in which case, the 1-sample reset would have no impact on performance – or alternatively, if the memory trace of recent COG samples encoded within the recurrent pathway was also being used as a predictive cue. Accordingly, we predicted that resetting the context layer units would not only impair performance of the SRN, but also that this interference effect would be greatest for the infants’ COG sequences.

It is important to stress in the 2- and 5-sample reset conditions, though, that this trace accumulates in a fashion that weights the memory toward COG samples that are more distal in time (i.e., past COG samples are not weighted equally). For example, in the 5-sample case, the first COG sample in a wave of five is effectively presented to the network as input (directly or indirectly) four times: once as the first COG sample, and then four more times as the trace of the sample cycles through the context units. By this logic, the fourth COG sample in the same wave of five is presented twice. Thus, the forgetting function provides a somewhat qualitative method for revealing whether or not sequential or temporal structure is present in infants’ COG image samples, but may not directly specify how those regularities are distributed over time. We return to this issue in the discussion and raise a potential strategy for addressing it.

## STIMULI

### OCCLUDED-ROD DISPLAY

During the collection of eye-tracking data (see below), the occluded-rod display was rendered in real-time. In order to convert this display into a sequence of still frames for the current simulation study, it was first captured as a video file (AVI format, 1280 × 1024 pixels, 30 fps), and then parsed by Matlab into still frames. A complete cycle of the rod’s movement, from the starting position on the far right, to the far left, and then back to the starting location, was extracted from the video and resulted in 117 frames (~3.5 s in real-time). Note that during video presentation, the dimensions of the occluded-rod display were 480 × 360 pixels, which was presented at the center of the monitor, surrounded by a black border. This border was subsequently cropped from the still-frame images, so that the occluded-rod display filled the frame. The gaze data obtained from infants were adjusted to reflect this cropping process; meanwhile, as we describe below, the simulated gaze data from the image-saliency and random-gaze models were obtained by presenting the cropped (480 × 360) occluded-rod displays to each model.

### OBSERVER GROUPS

#### Infants

Twelve 3-month-old infants (age, *M* = 87.7 days, SD = 12 days; 5 females) participated in the study. Infants sat on their parents’ laps approximately 60 cm away from a 76 cm monitor in a darkened room. Eye movements were recorded using the Tobii 1750 remote eye tracker. Before the beginning of each trial, an attention-getter (an expanding and contracting children’s toy) was used to attract infants’ gaze to the center of the screen. As soon as infants fixated the screen, the attention-getter was replaced with the experimental stimulus and timing of trials began. Each trial ended when the infant looked away for 2 s or when 60 s had elapsed. Note that all analyses described below were based on the eye-tracking data acquired during each infant’s first habituation trial (i.e., the occluded-rod display).

#### Image-saliency model

The saliency model was designed to simulate the gaze patterns of an artificial observer whose fixations and gaze shifts are determined by image salience, that is, by bottom-up visual features such as motion and light/dark contrast. In particular, the 117 still frames extracted from the occluded-rod display were transformed into a set of corresponding saliency maps by first creating four feature maps (tuned to motion, oriented edges, luminance, and color contrast, respectively) from each still-frame image, and then summing the feature maps into a saliency map. The sequence of 117 saliency maps was then used to generate a series of simulated fixations. We describe each of these processing steps in detail below.

***Feature maps.*** Each of the still-frame images was passed through a bank of image filters, resulting in four sets of feature maps: one motion map (i.e., using frame-differencing between consecutive frames), four oriented edge maps (i.e., tuned to 0°, 45°, 90°, and 135°), one luminance map, and two color-contrast maps (i.e., red–green and blue–yellow color-opponency maps). In addition, this process was performed over three spatial scales (i.e., to capture the presence of the corresponding features at high, medium, and low spatial frequencies), by successively blurring the original image and then repeating the filtering process [for detailed descriptions of the algorithms used for each filter type, refer to Itti et al. (1998) and Itti and Koch (2000)]. As a result, 24 total feature maps were computed for each still-frame image.

**FIGURE 2:**
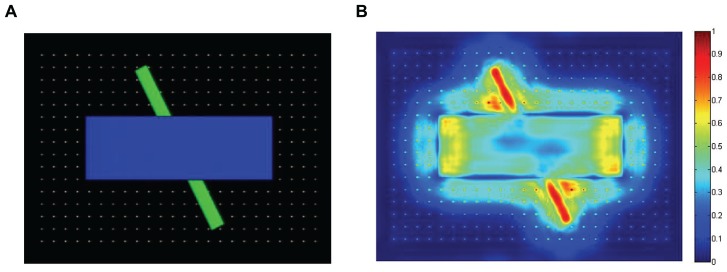
**Illustration of one of the still-frame images from the occluded-rod display (A), and the corresponding saliency map (B)**.

***Saliency maps.*** Each saliency map was produced by first normalizing the corresponding feature maps (i.e., by scaling the values on each map between 0 and 1), and summing the 24 maps together. For the next step (simulating gaze data), each saliency map was then downscaled to 40 × 30. These resulting saliency maps were then normalized, by dividing each map by the average of the highest 100 saliency values from that map. **Figure 2** illustrates a still-frame image from the occluded-rod display on the left, and the corresponding saliency map on the right.

***Simulated gaze data.*** Next, 12 sets of simulated gaze sequences were produced with the image-saliency model. Each set was yoked to the gaze data from a specific infant, and in particular, four dimensions of the infant and artificial-observer gaze sequences were equated: (1) the location (i.e., gaze point) of the first fixation, (2) the total number of fixations, (3) the duration of each fixation (i.e., dwell-time), and (4) the distance traveled between each successive fixation (i.e., gaze-shift distance).

At the start of the simulated trial, the image-saliency model’s initial gaze point was set equal to the location of the infant’s first fixation. The model’s gaze point was then held at this location for the same duration as the infant’s. For example, if the infant’s initial fixation was 375 ms, the model’s gaze point remained at the same location for 11 frames (i.e., 375 ms ÷ 33 ms/frame = 11 frames). In a comparable manner, each gaze shift produced by the image-saliency model was therefore synchronized with the timing of the corresponding infant’s gaze shift.

Subsequent fixation locations were selected by the image-saliency model by iteratively updating a fixation map for the duration of the fixation. The fixation map represents the difference between the *cumulative* saliency map (i.e., the sum of the saliency maps that span the current fixation) and a decaying inhibition map (see below). Note that the inhibition map served as an analog for an inhibition-of-return (IOR) mechanism, which allowed the saliency model to release its gaze from the current location and shift it to other locations on the fixation map.

Each trial began by selecting the initial fixation as described above. Next, the inhibition map was initialized to 0, and a 2D Gaussian surface was added to the map, centered at the current fixation point, with an activation peak equal to the value at the corresponding location on the saliency map. The Gaussian surface spanned a 92 × 92 pixel region, slightly larger than twice the size of a single COG sample (see COG Image Sequences, below). Over the subsequent fixation duration, activity on the inhibition map decayed at a rate of 10% per 33 ms. At the end of the fixation, the next fixation point was selected: (a) the fixation map was updated by subtracting the inhibition map from the saliency map (negative values were set to 0), (b) the top 500 values on the saliency map were chosen as potential target locations, and (c) the gaze-shift distance between the current fixation and each target location was computed. Finally, the target location with the gaze-shift distance closest to that produced by the infant (on the corresponding gaze shift) was selected as the next fixation location (any ties were resolved with a simulated coin-toss). The process continued until the model produced the same number of fixations as the corresponding infant (note that the sequence of 117 saliency maps were repeated as necessary).

#### Random-gaze model

The random-gaze model was designed as a control condition, to simulate the gaze pattern of an observer who scanned the occluded-rod display by following a policy in which all locations (at a given distance from the current gaze point) are equally likely to be selected. Thus, the gaze sequences were produced by the random-gaze model following the same four constraints as those for the image-saliency model (i.e., number and duration of fixations, gaze-shift distance, etc.), with the one key difference that upcoming fixation locations were selected at random (rather than based on image salience).

**FIGURE 3:**
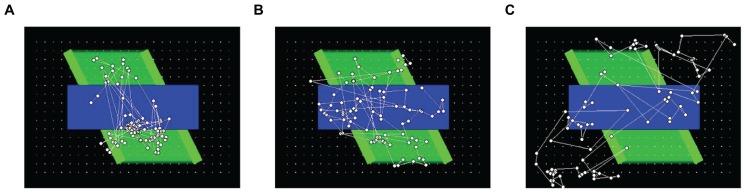
**Scanplot (sequence of fixation points) produced by one of the infants (A), together with the corresponding scanplots from the yoked image-saliency (B) and random-gaze models (C)**.

To help provide a qualitative comparison between typical gaze patterns produced by the three types of observers, **Figure 3** presents the cumulative scanplot from one of the infants (**Figure 3A**), as well as the corresponding scanplots from the image-saliency and random-gaze models that were yoked to the same infant (**Figures 3B,C**, respectively).

### SUMMARY STATISTICS

Prior to the training phase, we computed summary statistics for the three models, in order to verify that the yoking procedure resulted in comparable performance patterns for each yoked dimension. **Table 1** presents the mean summary statistics for the three observer groups (with standard deviations presented in parentheses). Note that the values presented in italics represent two of the four dimensions (i.e., fixation duration and gaze-shift distance) that were systematically equated between observer groups. In general, except where noted below, *post hoc* comparisons across the three observer groups revealed no significant differences. The first column presents the mean fixation duration (in milliseconds) for the infant, image-saliency, and random-gaze groups. The net difference between real and artificial observers was approximately 17 ms, and was presumably due to the fact while the infant data were measured continuously, the artificial observers were simulated in discrete time steps of 33.3 ms.

**Table 1:** Summary statistics as a function of observer group.

	Fixation duration	Saliency captured	Revisit rate	Fixation dispersion	Gaze-shift distance
Infant	*339.38 (96.03)*	0.66 (0.07)	0.23 (0.07)	78.55 (15.08)	*59.20 (18.82)*
Saliency	*356.19 (95.47)*	0.65 (0.03)	0.19 (0.11)	82.46 (18.68)	*60.36 (18.44)*
Random	*356.19 (95.47)*	0.47^*^ (0.05)	0.16 (0.08)	110.60^*^ (28.75)	*59.21 (18.82)*

* *p* < 0.01 (paired comparison vs. infant observer group). Standard deviation presented in parentheses; values in italics correspond to the two measures that were yoked across the three observer models.

The second column presents the mean saliency “captured” by each model, that is, the degree to which each group’s fixations were oriented toward regions of maximal saliency in the display. This was computed by projecting the gaze points produced by each of the observer groups on to the corresponding saliency maps, and then calculating the average saliency for those locations. Recall that values on the saliency maps were scaled between 0 and 1; the average saliency values from each group therefore reflected the proportion of optimal or maximal saliency captured by that group. There are two key results. First, the saliency model achieved an average of 0.65 saliency, indicating that – due to the constraint imposed on allowable gaze-shift distance – the model did not consistently fixate the most salient locations in the display. The second noteworthy finding is that infants’ gaze patterns captured a comparable level of saliency, that is, 0.66. As **Table 1** notes, the average saliency captured by the random observer group was significantly lower than the infant and image-saliency groups [both *t*s(22) > 8.46, *p*s < 0.001].

The third column presents the mean revisit rate for each observer group. Revisit rate was estimated by first creating a null frequency map (a 480 × 360 matrix with all locations initialized to 0). Next, for each fixation, the values within a 41 × 41 square (centered at the fixation location) on the frequency map were incremented by 1. This process was repeated for all of the fixations generated by an observer, and the frequency map was then divided by the number of fixations. For each observer, the maximum value from this map was recorded, reflecting the location in the occluded-rod display that was *most frequently* visited (as estimated by the 41 × 41 fixation window). The maximum value was then averaged across observers within each group, providing a metric for the peak proportion of fixations that a particular location in the occluded-rod display was visited, on average. As **Table 1** illustrates, a key finding from this analysis is that infants had the highest revisit rate (23%), while the two artificial observer groups produced lower rates.

The last two columns present kinematic measures of the gaze patterns. First, dispersion was computed by calculating the centroid of the fixations (i.e., the mean fixation location), then calculating the mean distance of the fixations (in pixels) from the centroid for each observer, and then averaging the resulting dispersion values for each group. As Figure, **Table 1** indicates, infants tended to have the least-disperse gaze patterns. Fixation dispersion in the image-saliency observer group did not differ significantly from the infant group, although it was significantly higher in the random-observer group [*t*(22) = 3.63, *p* < 0.01]. Finally, the fifth column presents the mean gaze shift distance (measured in pixels) for each group. Because this measure was yoked across groups, as expected, the artificial-observer groups produced mean gaze-shift distances that were comparable to the infants’ mean distance.

### COG IMAGE SEQUENCES

The final step, prior to training the model, was the process of mapping each set of gaze patterns into a sequence of COG image samples. This was accomplished by determining the frame number that corresponded to the start of each fixation, projecting the gaze point on to the resulting still-frame image, and then sampling a 41 × 41 pixel image, centered at that location. The dimensions of the COG sample were derived from the display size and infants’ viewing distance, and correspond to a visual angle of 1.8°, which falls within the estimated range of the angle subtended by the human fovea (Goldstein, 2010). In order to facilitate the training process, note that each of the COG samples was converted from color (RGB) to grayscale.

## MATERIALS AND METHODS

### MODEL ARCHITECTURE AND LEARNING ALGORITHM

Recall that our primary hypothesis was that infants’ COG sequences would be more easily learned by an SRN than the sequences from the two artificial-observer models. To evaluate this hypothesis, we trained a set of 3-layer Elman networks, with recurrent connections from the hidden layer back to the input layer (context units; Elman, 1990). In particular, this architecture implements a forward model, in which the current sensory input (plus a planned action) is used to generate a prediction of the next expected input (e.g., Jordan and Rumelhart, 1992). The complete model (including the training stimuli, network architecture, and learning algorithm) was written and tested by the first author (Schlesinger) in the Matlab programming environment.

The input layer of the SRN was composed of 2083 units, including 1681 units that encoded the grayscale pixel values of the current COG sample, 400 context units (which copied back the activity of the hidden layer from the previous time step), and two input units that encoded the x- and y-coordinates of the upcoming COG sample (normalized between 0 and 1). The input layer was fully connected to the hidden layer (400 hidden units, i.e., approximately 75% compression of the COG sample), which in turn was fully connected to the output layer (1681 units). The standard logistic function was used at the hidden and output layers to maintain activation values between 0 and 1; in addition, the bias terms were fixed to 0 for the hidden and output units.

An individual training trial proceeded as follows: given the selection of a COG sequence, the first COG sample in the sequence was presented to the SRN. For this first sample, the activation of the context units was set to 0.5. Activity in the network was propagated forward, resulting in the predicted next COG sample. This output was compared to the second COG sample in the sequence, and the root mean-squared error (RMSE) was calculated. Next, the standard backpropogation-of-error (i.e., backprop) learning algorithm was used to adjust the SRN’s connection weights (i.e., training was pattern-wise). The activation values from the hidden layer were then copied back to the input layer, and the second COG sample was presented to the SRN. This process continued until the second-to-last COG sample in the sequence was presented.

### TRAINING REGIME

A total of 10 training runs were conducted. At the start of each run, a single SRN was initialized with random connection weights between 0 and 1, which were then divided by the number of incoming units to the given layer (i.e., fan-in). This network was cloned 12 times, once for each of the infants. This duplication process ensured that any subsequent performance differences between SRNs during a run were due to the training samples unique to each infant, rather than to the initialization procedure.

Accordingly, each of the 12 SRNs was paired with one of the infants, and subsequently trained on the three COG sequences associated with that infant: the selected infant’s sequence, as well as the image-saliency and random-gaze sequences that were yoked to the same infant. A single training epoch was defined as a sweep through the three COG sequences. Order of observer type (i.e., infant, saliency, random) was randomized for each epoch. Pilot data collection indicated that the SRNs reached asymptotic performance, with a learning rate of 0.1, between 200 and 300 training epochs. As a result, each training run continued for 300 epochs.

In order to evaluate our second hypothesis – that resetting the activation of the context layer would have the largest interference effect on the infants’ COG sequences – we “paused” training every 10 epochs to test each of the SRNs. During the testing phase, learning was turned off and all connections were frozen in the SRN. Next, the SRN was tested by presenting the three COG sequences, four times each: (1) with recurrence functioning normally, and (2–4) with the activity of the context units reset to 0.5 every 1, 2, or 5 input steps, respectively.

## RESULTS

Two sets of planned analyses were conducted. First, we converted RMSE values into rank scores, and then compared the performance of the 12 SRNs as a function of mean rank of each observer group. In particular, this analysis focused on our predictions that the COG sequences from the infant group would have the highest mean ranking at the start of training, and that this difference would persist throughout the training period. The second analysis examined the influence of resetting the context-layer units on the SRNs’ performance, which allowed us to indirectly measure the presence of temporal dependencies in the COG sequences, between both adjacent samples as well as those as many as five samples apart.

**FIGURE 4:**
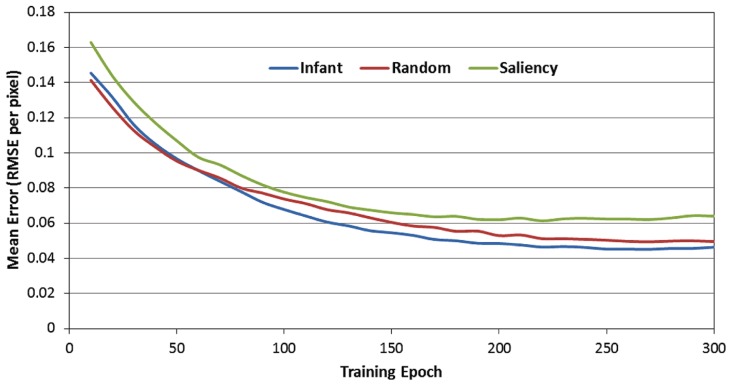
**Mean prediction error (MRSE per pixel) over the 300 training epochs, as a function of the three observer groups**.

**Figure 4** presents the RMSE produced by the 12 SRNs during the 300 training epochs, as a function of the observer group (i.e., infant, image-saliency, and random-observer models, respectively). Note that these data are pooled over the 12 SRNs and the 10 training runs. In addition, the RMSE values presented in **Figure 4** were those generated by the SRNs during the test phase, that is, in which learning was turned off every 10 epochs. As a result, these data reflect the performance of the SRNs while removing the transient effect of testing order (i.e., recall that the order of the observer groups during training was randomized across epochs).

There are two important trends suggested by **Figure 4**. First, the RMSE values produced by the image-saliency group remain consistently highest during training. Second, there is an early “trade-off” between the infant and random-gaze groups, which eventually results in a stable difference, favoring the infant group. In order to determine whether these trends were statistically reliable, we first converted the RMSE values into ranks. In particular, for each epoch, the RMSE for the three observer groups were sorted in ascending order, and assigned the corresponding rank (i.e., 1, 2, or 3). As before, ranks were then averaged over the 12 SRNs and 10 training runs.

**FIGURE 5:**
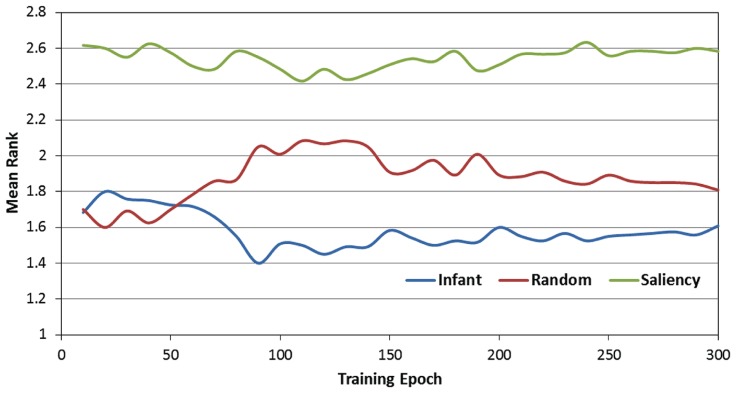
**Mean rank scores over the 300 training epochs, as a function of the three observer groups**.

**Figure 5** presents the rank-transformed performance data. (Note that in describing these data, we adopt the convention that the rank of 1 is treated as “highest” while the rank of 3 is the “lowest.” In other words, a higher average rank corresponds to a lower RMSE). In order to compare the three observer groups, a 2-way ANOVA was conducted with epoch and observer group as the two factors. As expected, there was a main effect of observer group [*F*(2,357) = 124.24, *p* < 0.001]. We examined this effect with planned paired comparisons between the three groups (using Bonferroni corrections), which also confirmed our prediction: specifically, the infant observer group had significantly higher overall mean rank than the image-saliency and random-gaze groups. However, these findings were qualified by a significant epoch × observer group interaction [*F*(58,10353) = 6.48, *p* < 0.001]. As **Figure 5** indicates, near the start of training, the infant and random-gaze groups had similar ranks; in contrast, a large, stable difference between the two groups emerged after approximately 50 epochs.

In order to examine this interaction, we conducted a *post hoc* analysis by first dividing training time into two phases (0 to 50 and 60 to 300 epochs). We then repeated the previous 2-way ANOVA for each phase (i.e., epoch × observer group), including comparisons between the three observer groups. This analysis revealed that while there was no significant difference between the infant and random-gaze groups during the first 50 epochs (*p* = 0.64), the infant group averaged a significantly higher rank than the random-gaze group during the remaining 250 epochs (*p* < 0.005). In particular, these results confirm our prediction that the infant observer group would be ranked highest at the start of training, albeit after an initial period of equivalent performance in two of the three groups. In addition, the stability of this pattern for the remainder of the training phase also provides support for our prediction that the infant observer group would maintain the highest rank throughout training.

**FIGURE 6:**
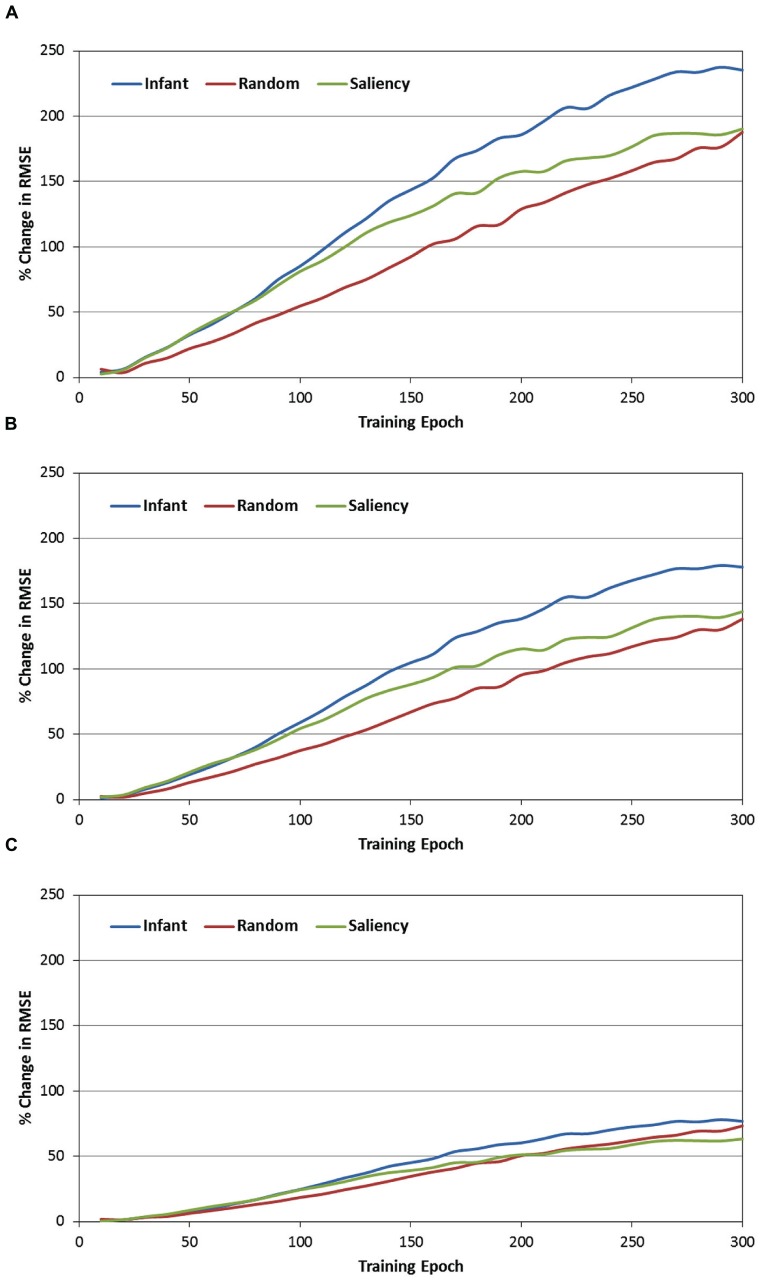
**Percent change in the MRSE during testing of the three observer groups, while resetting the recurrent layer units after every sample (A), every other sample (B), and every five samples (C)**.

The second set of analyses focused on the role of the context layer in the SRN architecture, and more specifically, on the question of whether periodically resetting the activity of this layer during training would disrupt performance. In order to address this question, recall that during each test phase, each of the SRNs was not only tested under canonical conditions (e.g., full recurrence; see **Figure 4**), but also under three conditions in which the context layer was reset (i.e., all values were set to 0.5) after every 1, 2, or 5 training samples. Because it was anticipated that resetting the context layer would produce an increase in prediction errors, RMSE difference scores were therefore computed between each of the reset conditions and the canonical condition. These difference scores were then transformed into percent-change scores, relative to the canonical condition (that is, percent increase in the RMSE due to resetting the context layer). **Figure 6** presents the resulting percent-change values for each of the observer groups, within the three reset conditions (i.e., 6A = every sample, 6B = every two samples, and 6C = every five samples, respectively).

There are three primary findings from this analysis. First, a consistent pattern observed across the three observer groups and reset conditions is that the percent change of the RMSE starts near 0 at the beginning of training. However, for all groups and conditions, this value quickly increases, reflecting a progressively greater impact of resetting the context layer over training time. For example, **Figure 6A** illustrates that by the end of training, resetting the context layer after each COG sample results in approximately a 200% increase in the RMSE, on average for the three observer groups. Second, there is a positive association between the reset frequency and the percent increase in RMSE. In other words, resetting the context layer after every sample produced a larger interference effect than resetting every two samples, and likewise for resetting every five samples.

Third, we conducted a 2-way ANOVA for each of the reset conditions, again with epoch and observer groups as the two factors. This comparison revealed a significant epoch × observer group interaction for all three reset conditions [all *F*s(58, 10353) > 3.87, *p*s < 0.001]. In general, as **Figure 6** illustrates, this interaction reflects the tendency for percent-change scores to begin near 0 for each of the observer groups, and then subsequently increase at different rates over training time. We pursued this interaction by dividing training time into three blocks of epochs (i.e., 0–100, 100–200, and 200–300 epochs), and then conducting a simple-effects test of observer group for each of the three blocks. Two consistent findings emerged from this test. First, across each of the three training blocks and two of the three reset conditions, the percent increase of the RMSE in the infant group was significantly higher than in the random-gaze group [all *t*s(238) > 2.79, *p*s < 0.02]. The only exception to this result was in the condition where the context layer was reset every five samples, during the final block of epochs; in this case, the infant and random-gaze groups did not significantly differ. Second, a significant difference between the infant and saliency groups was not present during the first two blocks of epochs (i.e., through epoch 200). However, by the third block of epochs, the percent increase in RMSE in the infant group was significantly higher than in the saliency group, for all three reset conditions [all *t*s(238) > 2.38, *p*s < 0.05]. Taken together, these findings collectively support our prediction that resetting the context-layer activation values would have the largest interference effect on the infants’ COG sequences.

## DISCUSSION

The current simulation study focused on two goals. First, we sought to demonstrate that our previous gaze-sequence learnability findings, from an infant free-viewing task (Schlesinger and Amso, 2013), would generalize and extend to a task that was specifically designed to study object perception in young infants. Second, we not only implemented several key improvements in our model, but also modified the training and testing procedure to allow us to assess whether learnability of the infants’ COG samples was due, at least in part, to the presence of sequential dependencies between both adjacent and non-adjacent training samples.

The results were consistent with each of our four hypotheses. First, we predicted that infants’ COG sequences would be learned first by the 12 SRNs. We assessed this prediction by converting each observer group’s error scores into ranks and then analyzing the respective ranks over 300 epochs of training time. As we predicted, the infant group eventually established a significant advantage over the other two observer groups. Unexpectedly, however, this advantage did not appear at the onset of training. Instead, the average ranks of the infant and random-gaze groups were comparable for the first 50 epochs of training. One potential explanation for this early similarity of performance in the two observer groups is that there was a higher initial “learning cost” associated with the infant group, due to the (presumed) presence of temporal dependencies in their COG sequences, which ostensibly required additional time for the SRNs to detect and exploit (through the context layer). Second, we also predicted that this advantage would persist and remain stable across the remaining time. Again, the results supported our prediction.

Our third and fourth predictions focused on whether the success of the SRN architecture in learning the infants’ COG sequences benefited from the (presumed) presence of temporal or sequential dependencies embedded within the infants’ COG training samples. Luckily, the use of the random-gaze model provides a critical role in addressing this question, as the gaze sequences from this model were specifically produced with a stochastic procedure (although it should be noted that the selection of each gaze point was constrained by a fixed gaze-shift distance rule). As a result, we can thus assume that there were no *a priori* regularities or dependencies within the random-gaze model’s COG sequences, other than those broadly present in the display itself (e.g., the baseline probability of fixating the background, or the occluding screen, at random).

We therefore predicted that disrupting information flow within the recurrent pathway of the network by periodically resetting the context layer would increase the overall errors produced by the SRNs. Indeed, across all three observer groups we observed significant increases in the SRN prediction errors when the recurrent layer was reset. Our last prediction was that the interference effect would be greatest for the infants’ COG sequences, and as **Figure 6** illustrates, this prediction was confirmed as well.

Further inspection of **Figure 6** may offer three additional insights. First, as we suggested above, the gaze sequences produced by the random-gaze model should include minimal (if any) sequential structure. Nevertheless, note that – like the other two observer groups – the interference effect increased with training time in the random-gaze group. This trend provides a statistical baseline for estimating the contribution of the context layer for prediction learning on the current task, as the training sequences from the random-gaze model were ostensibly sequentially independent. We can therefore estimate the presence of any additional structure embedded within infants’ COG sequences by subtracting the RMSE change values produced by the random-gaze model. For example, in the first reset condition (i.e., reset after every sample) and pooling over training time, the overall difference in RMSE change between the infant and random-gaze groups is 42%. This value provides an important clue toward understanding the function of infants’ object-directed gaze behavior, as it demonstrates that infants’ gaze sequences are significantly more structured than sequences produced by chance, and that this embedded sequential structure also provides a measurable advantage to an active observer that is learning to forecast or predict the content of upcoming fixations.

An additional insight offered by manipulating the context layer is reflected by the regular order of performance observed across the three observer groups. In particular, note that the interference effect was consistently lowest in the random-gaze group, highest in the infant group, and midway between the two in the image-saliency group. This finding suggests that the simple strategy of orienting toward relatively high-saliency regions in the occluded-rod display is sufficient to generate statistically reliable temporal structure in the COG sequences.

Finally, a third insight suggested by these findings is that image-saliency may provide, at best, a partial account for how infants’ gaze patterns are structured over time and space. In particular, our previous work has demonstrated that a saliency-based model captures several global-level features of infants’ gaze patterns, such as the frequency of fixations toward the rod segments, as well as individual differences in the rate of rod fixations between infants (Amso and Johnson, 2006; Schlesinger et al., 2007, 2012). In addition, our current model provides two additional pieces of evidence that also implicate the role of image saliency. First, as **Table 1** indicates, the infant and image-saliency groups fixated regions of the occluded-rod display that were on average nearly equal in salience. Second, as **Figure 6** illustrates, resetting the context layer had a comparable effect on the infant and image-saliency groups during the first 75–80 epochs of training (the same pattern was also consistent across the three reset conditions).

However, after approximately 80 epochs, the interference effect continued to increase at a faster rate in the infant group. One potential interpretation for this pattern is that, due to similar levels of saliency in the infants’ and image-saliency models’ COG samples, the SRNs “focused” during early learning on saliency-related features in the input (e.g., luminance contrast) as a predictive cue. In contrast, the random-gaze model fixated salient locations less frequently (i.e., 42% of maximal salience, vs. 66 and 65% in the infant and image-saliency models, respectively), and as a result, recurrent feedback in the SRN had less impact on prediction learning for the sequences from this observer model. If this reasoning is correct, it suggests that the subsequent performance split between the infant and image-saliency models was presumably due to additional temporal structure – beyond that provided by saliency – in the infants’ sequences, which the SRNs continued to learn to detect and exploit. To put the point concisely: while infants and the image-saliency model fixated (on average) equally-salient regions in the occluded-rod display, we are proposing that it was *the particular temporal order in which infants scanned salient regions of the display* that provided an additional predictive cue to the SRNs. We are currently exploring computational strategies for teasing apart these spatial and temporal cues, and isolating their influence on the prediction-learning process.

Two key issues remain unaddressed by our work thus far. First, it is important to note that our use of the SRN architecture, as well as our manipulation of the context layer, provide a somewhat indirect method for identifying sequential structure in infants’ COG samples. In general, this strategy tells us that temporal structure is present and it also provides a method for quantifying the interference caused by periodically resetting the context units, but it does not directly identify the visual features detected by the SRN, not does it specify how variation in these cues over time (i.e., correlations between successive COG samples) improves the outcome of sequence learning. An additional limitation of the reset method, which we noted in the introduction, is that the samples that are processed before a reset occurs do not contribute equally to the memory trace that accumulates in the recurrent pathway (i.e., distal samples are weighted more than recent samples).

There are several strategies available to address these issues. For example, alternative analytical methods (e.g., principal-component or clustering analysis of the hidden layer activations) as well as alternative modeling architectures and learning algorithms (e.g., Kohonen networks, Kalman filters, etc.) may provide additional insights. We are also currently exploring the strategy of constructing artificial gaze sequences in which we strictly control the statistical dependencies over time (e.g., alternating gaze between 2, or 3, or 4 narrowly defined regions in an image). Ideally, this will allow us to examine the influence of resetting the context layer versus learning/detecting temporal dependencies that vary in their duration over time. A related limitation of the modeling strategy we have employed here is that the SRNs were trained over multiple repetitions of the same COG sequences. In particular, this repetition provides an important learning cue to the SRNs, independent of the temporal structure embedded within the COG sequences. One way to address this issue is to employ a “leave-out” training regime, in which a subset of training patterns are set aside and reserved for testing the model.

Second, we should also note that our current simulation study focused exclusively on infants’ first trial during the perceptual-completion task. An open question is whether infants’ scanning patterns change systematically over subsequent trials (e.g., do rod fixations increase?), and if so, what effect if any will such changes have on the predictability of the COG sequences that are produced during later trials? Our intuition is that if infants’ gaze patterns during later trials are less variable (e.g., as estimated by our dispersion measure), *their COG sequences will become more predictable* (due to greater similarity between sequences). In addition, recall that after habituating to the occluded-rod display, infants then view the solid-rod and broken-rod test displays (**Figure 1**). Therefore, a related question is whether predictability of the COG sequences will increase or decrease during the test trials, and in particular, whether it will vary across the two display types. Answering these questions is essential to understanding the role of visual prediction-learning during the development of object perception.

We now return to the issue of early object-perception development in young infants. Our work has not only implicated the role of active visual scanning as an essential skill for object perception (Johnson et al., 2004; Amso and Johnson, 2006), but also demonstrated how this skill can emerge developmentally through interactions between the parietal and occipital cortex (Schlesinger et al., 2007). Recent work has also implicated visual prediction-learning as a complementary mechanism that may also support object perception (Schlesinger et al., 2011; Schlesinger and Amso, 2013). Our current findings help to integrate these ideas into a coherent developmental mechanism, by not only demonstrating that sequential structure is present within infants’ time-ordered gaze patterns, but also that this structure is manifest across both complex, naturalistic displays as well as the relatively simplified ones that are used to investigate object perception in the laboratory. An additional important insight from both our recent behavioral and modeling work is that perceptual salience is likely a necessary, though not sufficient cue for driving visual scanning and object exploration in young infants (Schlesinger and Amso, 2013; Amso et al., 2014). We are optimistic that future work on this question will help to identify the other cues and sources of temporal structure that infants are learning to detect and exploit.

Finally, we conclude by noting that our modeling approach has the potential to offer two important innovations for the study of perceptual development in infants. First, our current strategy is to analyze infants’ COG sequences offline, that is, *after they have been produced*. Thus, one of our long-term goals is to design an architecture that can accurately forecast infants’ upcoming fixations *before they are produced*. One application of this forecasting technique would then be to manipulate the features or properties of the gaze destination before the infant gazed at that location, as a way of gauging their sensitivity to those features (i.e., a kind of gaze-contingent change-blindness paradigm). Second, we have previously observed variation across infants at the same age with visual displays such as the perceptual-completion task (e.g., Amso and Johnson, 2006). We are now excited to see if infants’ performance on the perceptual-completion task will correlate with the relative learnability of the COG sequences they produce during the occluded-rod display, which would provide further support for the idea that individual differences in information pick-up have a fundamental effect on the development of object perception.

## Conflict of Interest Statement

The authors declare that the research was conducted in the absence of any commercial or financial relationships that could be construed as a potential conflict of interest.

## References

[B1] Amso D. Haas S. Markant J. (2014). An eye tracking investigation of developmental change in bottom-up attention orienting to faces in cluttered natural scenes. *PLoS ONE* 9:e85701 10.1371/journal.pone.0085701 PMC3899069 24465653

[B2] Amso D. Johnson S. P. (2006). Learning by selection: visual search and object perception in young infants. *Dev. Psychol.* 42 1236–1245 10.1037/0012-1649.42.6.1236 17087555

[B3] Banks M. S. Salapatek P. (1978). Acuity and contrast sensitivity in 1-, 2-, and 3-month-old human infants. *Investigat. Ophthalmol. Vis. Sci.* 17 361–365 640783

[B4] Bronson G. (1982). *The Scanning Patterns of Human Infants: Implications for Visual Learning*. Norwood, NJ: Ablex

[B5] Bronson G. (1991). Infant differences in rate of visual encoding. *Child Dev.* 62 44–54 10.2307/1130703 2022137

[B6] Clohessy A. B. Posner M. I. Rothbart M. K. Vecera S. P. (1991). The development of inhibition of return in early infancy. *J. Cogn. Neurosci.* 3 345–350 10.1162/jocn.1991.3.4.345 23967814

[B7] Dannemiller J. L. (2000). Competition in early exogenous orienting between 7 and 21 weeks. *J. Exp. Child Psychol.* 76 253–274 10.1006/jecp.1999.2551 10882475

[B8] Dragoi V. Sur M. (2006). Image structure at the center of gaze during free viewing. *J. Cogn. Neurosci.* 185 737–748 10.1162/jocn.2006.18.5.737 16768374

[B9] Elman J. L. (1990). Finding structure in time. *Cogn. Sci.* 14 179–211 10.1207/s15516709cog1402_1

[B10] Elman J. L. (1993). Learning and development in neural networks: the importance of starting small. *Cognition* 48 71–99 10.1016/0010-0277(93)90058-4 8403835

[B11] Fantz R. L. (1956). A method for studying early visual development. *Percept. Mot. Skills* 6 13–15 10.2466/pms.1956.6.g.13

[B12] Goldstein B. (2010). *Sensation and Perception*. Belmont, CA: Wadsworth

[B13] Haith M. M. (1980). *Rules that Babies Look by: The Organization of Newborn Visual Activity*. Hillsdale, NJ: Erlbaum

[B14] Haith M. M. (1994). *The Development of Future-Oriented Processes*. Chicago: University of Chicago Press

[B15] Itti L. Koch C. (2000). A saliency-based search mechanism for overt and covert shifts of visual attention. *Vision Res.* 40 1489–1506 10.1016/S0042-6989(99)00163-7 10788654

[B16] Itti L. Koch C. Niebur E. (1998). A model of saliency-based visual-attention for rapid scene analysis. *IEEE Trans. Pattern Analysis Mach. Intell.* 20 1254–1259 10.1109/34.730558

[B17] Johnson S. P. Amso D. Slemmer J. A. (2003). Development of object concepts in infancy: evidence for early learning in an eye-tracking paradigm. *Proc. Natl. Acad. Sci. U.S.A.* 100 10568–10573 10.1073/pnas.1630655100 12939406 PMC193601

[B18] Johnson S. P. Aslin R. N. (1995). Perception of object unity in 2-month-old infants. *Dev. Psychol.* 31 739–745 10.1037/0012-1649.31.5.739

[B19] Johnson S. P. Slemmer J. A. Amso D. (2004). Where infants look determines how they see: eye movements and object perception performance in 3-month-olds. *Infancy* 6 185–201 10.1207/s15327078in0602_3 33430533

[B20] Jordan M. J. Rumelhart D. E. (1992). Forward models: supervised learning with a distal teacher. *Cogn. Sci.* 16 307–354 10.1207/s15516709cog1603_1

[B21] Kellman P. J. Spelke E. S. (1983). Perception of partly occluded objects in infancy. *Cognit. Psychol.* 15 483–524 10.1016/0010-0285(83)90017-8 6641127

[B22] Kienzle W. Franz M. O. Schölkopf B. Wichmann F. A. (2009). Center-surround patterns emerge as optimal predictors for human saccade targets. *J. Vis.* 9 1–15 10.1167/9.5.7 19757885

[B23] Mohammed R. A. A. Mohammed S. A. Schwabe L. (2012). BatGaze: a new tool to measure depth features at the center of gaze during free viewing. *Brain Inform.* 7670 85–96 10.1007/978-3-642-35139-6_9

[B24] Saffran J. R. Aslin R. N. Newport E. L. (1996). Statistical learning by 8-month-old infants. *Science* 274 1926–1928 10.1126/science.274.5294.1926 8943209

[B25] Schlesinger M. Amso D. (2013). Image free-viewing as intrinsically-motivated exploration: estimating the learnability of center-of-gaze image samples in infants and adults. *Front. Psychol.* 4:802 10.3389/fpsyg.2013.00802 PMC3813899 24198801

[B26] Schlesinger M. Amso D. Johnson S. P. (2007). The neural basis for visual selective attention in young infants: a computational account. *Adapt. Behav.* 15 135–148 10.1177/1059712307078661

[B27] Schlesinger M. Amso D. Johnson S. P. (2011). “Increasing spatial competition enhances visual prediction learning,” in *Proceedings of the First Joint IEEE Conference on Development and Learning and on Epigenetic Robotics* eds Cangelosi A. Triesch J. Fasel I. Rohlfing K. Nori F. Oudeyer P.-Y. Schlesinger M. Nagai Y. (New York: IEEE) 1–6

[B28] Schlesinger M. Amso D. Johnson S. P. (2012). Simulating the role of visual selective attention during the development of perceptual completion. *Dev. Sci.* 15 739–752 10.1111/j.1467-7687.2012.01177.x 23106728 PMC4101467

[B29] Slater A. (2002). Visual perception in the newborn infant: issues and debates. *Intellectica* 34 57–76

[B30] von Hofsten C. (2010). Prospective control: a basic aspect ofaction development. *Hum. Dev.* 36 253–270 10.1159/000278212

